# Investigation of technical quality of antenatal and perinatal services in a nationally representative sample of health facilities in Nepal

**DOI:** 10.1186/s13690-022-00917-z

**Published:** 2022-07-04

**Authors:** Resham B. Khatri, Jo Durham, Yibeltal Assefa

**Affiliations:** 1 School of Public Health, Faculty of Medicine, the University of Queensland, Brisbane, Australia; 2 Health Social Science and Development Research Institute, Kathmandu, Nepal; 3 School of Public Health and Social Work, Queensland University of Technology, Brisbane, Australia

**Keywords:** Technical quality, Health system readiness, Quality care, Determinants, Antenatal care, Perinatal services, Health facilities, Nepal

## Abstract

**Background:**

Access to routine antenatal and perinatal services is improved in the last two decades in Nepal. However, gaps remain in coverage and quality of care delivered from the health facilities. This study investigated the delivery of technical quality antenatal and perinatal services from health facilities and their associated determinants in Nepal.

**Methods:**

Data for this study were derived from the Nepal Health Facility Survey 2015. The World Health Organization's Service Availability and Readiness Assessment framework was adopted to assess the technical quality of antenatal and perinatal services of health facilities. Outcome variables included technical quality scores of i) 269 facilities providing antenatal services and ii) 109 facilities providing childbirth and postnatal care services (perinatal care). Technical quality scores of health facilities were estimated adapting recommended antenatal and perinatal interventions. Independent variables included locations and types of health facilities and their management functions (e.g., supervision). We conducted a linear regression analysis to identify the determinants of better technical quality of health services in health facilities. Beta coefficients were exponentiated into odds ratios (ORs) and reported with 95% confidence intervals (CIs). The significance level was set at *p*-value < 0.05.

**Results:**

The mean score of the technical quality of health facilities for each outcome variable (antenatal and perinatal services) was 0.55 (out of 1.00). Compared to province one, facilities of Madhesh province had 4% lower odds (adjusted OR = 0.96; 95%CI: 0.92, 0.99) of providing better quality antenatal services, while health facilities of Gandaki province had higher odds of providing better quality antenatal services (aOR = 1.05; 95% CI: 1.01, 1.10). Private facilities had higher odds (aOR = 1.13; 95% CI: 1.03, 1.23) of providing better quality perinatal services compared to public facilities.

**Conclusions:**

Private facilities provide better quality antenatal and perinatal health services than public facilities, while health facilities of Madhesh province provide poor quality perinatal services. Health system needs to implement tailored strategies, including recruiting health workers, supervision and onsite coaching and access to necessary equipment and medicine in the facilities of Madhesh province. Health system inputs (trained human resources, equipment and supplies) are needed in the public facilities. Extending the safe delivery incentive programme to the privately managed facilities could also improve access to better quality health services in Nepal.

**Supplementary Information:**

The online version contains supplementary material available at 10.1186/s13690-022-00917-z.

## Background

Sustainable development goal three (SDG3) has targets to reduce the Maternal Mortality Ratio (MMR) and Neonatal Mortality Rate (NMR) to 70 per 100,000 live births [1] and 12 per 1,000 live births, respectively [2, 3]. Reaching the SDG3 targets, however, requires accelerated progress in expanding access to quality antenatal and perinatal care, especially in Sub-Saharan Africa and South Asia, which constitute 86% of maternal mortalities [2, 3] and more than 90% of neonatal deaths globally [4]. While there have been significant gains in coverage of essential antenatal and perinatal services, this has not translated into significant progress in reducing the MMR and NMR within these regions [5, 6]. For example, in India, the institutional delivery rate increased from 39 to 80% from 2005 to 2015 [7], partly due to the implementation of maternity incentive program (Janani Surkshya Yojana) [8]. However, in the same period, the NMR only reduced from 37 to 28 per 1000 live births [9]. Similarly, in Ethiopia, the institutional delivery rate increased from 16 to 41% from 2011 to 2016 [10], but improvements in the MMR reduction were modest (reduced from 597 to 446 per 100,000 live births) [1]. Regional and national averages also mask disparities in maternal and newborn health (MNH) outcomes based on socioeconomic status of populations. For example, an analysis of multi-country demographic health survey data showed a modest decline in the NMR among low wealth status groups compared to high wealth status groups, despite similar increases in access to MNH services, including institutional delivery [11]. These figures suggest increasing coverage of services on its own, is not enough, and more attention is needed to the quality of care that women receive. The importance of focusing on coverage and service quality is also recognised in recent global health plans and strategies, including the Every Newborn Action Plan [12], the Global Strategy for Women's, Children's and Adolescents' Health 2016–2030 [13], and Strategies Towards Ending Preventable Maternal Mortality [14].

Along with other countries in South Asia, over the last two decades, Nepal has made good progress in improving access to routine MNH services around antenatal (conception to before childbirth) and perinatal (22 weeks of pregnancy to 7^th^ day after childbirth) period [15]. For instance, institutional delivery increased from 18% to 59% between 2006 and 2016. The reduction in MMR and NMR, however, has been moderate (MMR reduced from 281 (per 100,000 live births) to 259, and the NMR decreased from 33 (per 1000 live births) to 22). The most cited reasons for the slow reduction of the MMR and NMR are inequities in access to health services among socioeconomically disadvantaged groups and for those in geographically remote areas [16–18]. Another reason relates to the poor access to good quality of antenatal and perinatal services [19]. Therefore, recent policies, such as the Nepal Health Sector Strategy (2015–2020) [20], the Nepal Health Policy 2019 [21] and the Nepal Safe Motherhood and Newborn Health Road Map 2030 highlight equity in access to better quality health services as the health system's overarching principle [22].However, to track the implementation of these policy provisions, monitoring of health system readiness and delivery of quality MNH services is required.

To date, most studies in Nepal examining quality of care have focused on client satisfaction [23–26]. While important, client satisfaction is subjective and contextual, based on individual and community preferences and understandings of quality of care. Quality of care is multidimensional and complex and includes subjective and objective measures. However, assessing objective or technical quality measures of care is often compounded by a lack of input data [27]. At health facilities quality of care constitutes two components: quality of health services available (health system readiness or input quality) and quality of care delivered (process quality or technical or clinical quality) [28–30]. Health system readiness or input quality (also considered structural quality) is the condition that health facilities are equipped with necessary human resources, equipment, medicines, and infrastructure, guidelines for any health services. In contrast, process quality is actual delivery of health services or technical interventions [31]. For example, if any health facility has better input quality for antenatal services, that means that facility has the necessary standards as defined by the national antenatal care guideline. While if any health facility delivered better quality antenatal care services, pregnant women could receive recommended antenatal interventions when she visited that facility [32]. In other words, for better technical quality antenatal and perinatal services, input factors provide the prerequisite environment for delivering clinical interventions for healthy pregnancies and babies [31]. The process quality relates to health services delivery and comprises technical and social quality (or perceived services quality) [33]. Technical quality constitutes the delivery of technical interventions, while social quality is about clients' satisfaction, including dignity and respectful care.

### Nepal’s Health system and policy context for antenatal and perinatal health

**Fig. 1:**
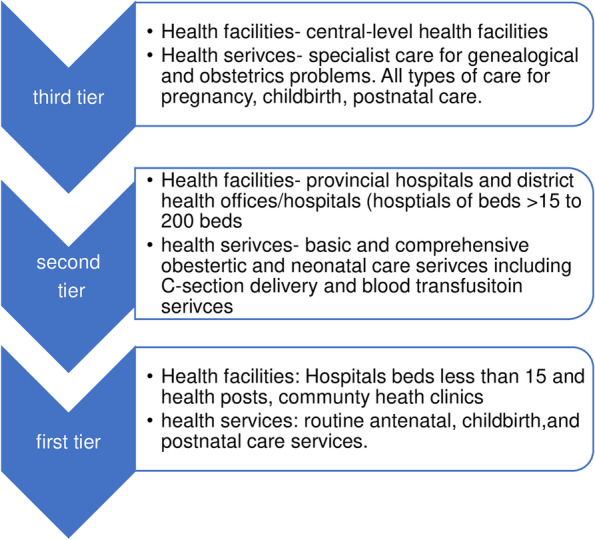
Three-tiered health system and services for maternal and newborn health in Nepal

Nepal has a three-tier federal health system in line with the governance system (Fig. 1): federal, provincial, and local or municipal governments [34]. Nepal's current federal health system has decentralised the resources and authorities to provincial and local governments [35]. All central-level health facilities are governed by the federal government, which refers to the third tier of the health system. These facilities provide tertiary care including specialist care of genealogical and obstetrics problems. The second tier of health system includes provincial hospitals and district health offices/hospitals and are managed by the provincial governments. These facilities provide specialised care, including cesarian-section and blood transfusion services. The Ministry of Social Development of each province governs the provincial health system. Local governments or municipal governments govern the first tier of health system. Health facilities under the first tier of health systems include community (community health clinics, outreach clinics), and ward level facilities (health posts), primary health care centers, and hospitals with less than15 beds. These facilities provide essential routine antenatal and perinatal services. Total of 753 municipalities (with 6743 wards) have their health sections that govern their ward-level health facilities.

Over the last three decades in Nepal, several policy and programs played a significant role in improving access to antenatal and perinatal services. Nepal has a major policy shift in health care, including implementing Nepal’s first National Health Policy in 1991 [36]. The detailed trajectory of health policy, mainly MNH policies and programs, are available in supplementary Table S1 (adapted from [37] p:171–173). The first National Health Policy provisioned one facility in each ward (then Village Development Committee, the lowest administrative unit). Since 1997, several health policies have been formulated, such as the Sexual and Reproductive Health Policy (1997), Neonatal Health Strategy (2004), National Safe Motherhood and Newborn Health Long Term Plan (2002–2017), Safe Abortion Policy (2002), Safe Delivery Incentive Program (2005) [38–40]. Some MNH programs implemented include the Community-Based Newborn Care Program (2007), Integrated Management of Neonatal and Childhood Illness (2014), and Free Newborn Care Program (2015 [41].

**Fig. 2:**
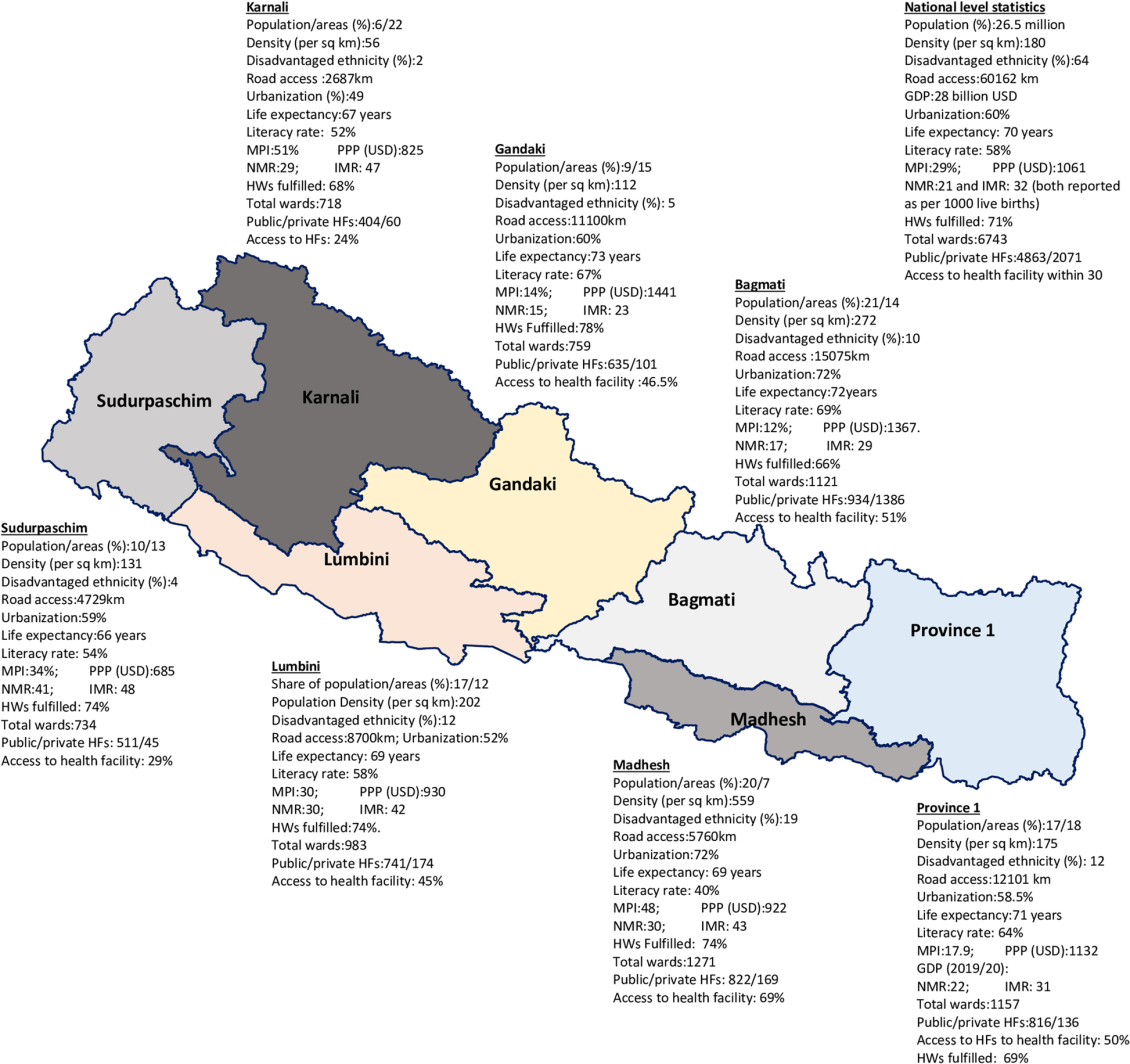
Provincial map of Nepal with health and socioeconomic indicators, 2020*

The federal republic constitution of Nepal (2015) has provisioned basic/essential health services as the fundamental rights of people, and should be provided through public funding [34].Nepal’s health care delivery system is mixed. Private providers are dominant in secondary and tertiary health services and are located in urban areas [39]. The public health system provides basic and secondary health care services in urban, rural, and regional areas. Two-thirds of private facilities, such as hospitals are urban centred and are mainly in Bagmati province (the federal Capital of Nepal) (Fig. 2). Basic health care services are available in public facilities through tax-funded public health programs. At the same time, health services beyond the basic health service package require to pay for services through out-of-pocket expenditure even in public facilities. The care cost in private facilities is high, including basic health services such as routine antenatal and perinatal services.

The National Health Insurance Program (NHIP) was rolled out in 2016 and offered to cover the cost of care in some public facilities (and very few contracted private health facilities also) (up to 1000 USD annually) beyond essential/basic health services. However, the program has experienced challenges in implementation, including low enrollment and high dropout in the renewal of the membership [42]. Furthermore, while the scheme is intended to increase financial protection by promoting pre-payment and risk pooling in the health sector, it is only applicable in government-designated health facilities. Only 440 facilities are accredited as providers of NHIP insured persons, while enrolment under this program is minimal (only 11% of the total population) [43]. The government policy also encourages private facilities to contract in this program, where public facility acts as the first point of contact, and referral can be made to contracted private facilities[44]. However, the participation of private facilities is low, and therefore, there are no financial risk protection mechanisms for health care bills paid at private facilities [45]. Private facilities can result in high out-of-pocket expenditure, including essential health services in the absence of social health insurance, and a lack of private health insurance [46]. Even in private facilities, there is no uniformity in the cost of care, and these private facilities charge a high cost of care, claiming better quality of care they provide. However, there is no formal mechanism to monitor the quality of care, either through routine system or periodic assessment.

Nepal's current health management information system (HMIS) lacks data on inputs, service availability, facility readiness and technical quality of care [39], presenting challenges in assessing and monitoring the quality of health services. The Nepal Health Facility Survey (NHFS) 2015 collected information from multiple sources, including health facility inventory, observations, and client exit interviews [32]. Further processing of the survey data could help identify and monitor the quality of care delivered in facilities. Such an analysis could complement client satisfaction data and provide a more robust assessment of the technical quality of antenatal and perinatal services. This study aimed to investigate the delivery of technical quality of antenatal and perinatal services from health facilities and their associated actors in Nepal. The findings of this study are important in providing policy-makers with the information needed to design appropriate programs for quality improvement in Nepal. In addition, the study provides insights on how national health facility survey data can be used to assess technical quality of care at the health facility level.

## Methods

### Study design and setting

This study used data derived from the NHFS 2015 [32], which was a cross-sectional study based on a representative sample of the main types (such as hospitals, primary health care centers, health posts and clinics) of facilities in Nepal. The 2015 NHFS was based on generic designs and modules developed by the Measure Demographic Health Survey, ICF Macro, Maryland. In addition, the core tools were revised and modified to the country context and aligned with WHO’s Service Availability and Readiness Assessment (SARA) manual [28].

Figure 2 is the map of federal Nepal showing important province-specific health and socioeconomic indicators. Out of seven provinces, Karnali province is the remotest province with low socioeconomic indicators; it has difficult geographic terrain and has mountains and hills in almost all parts. While Madhesh province lies in the southern Terai (plain) region and bordering with India, it also has a low status of health and development indicators [47]. In this province, most of the populations belong to the disadvantaged population (Madhesi) group. As a result, most health and socioeconomic indicators are lower compared to other provinces [47, 48].

 *Source of Fig. 2: Prepared by the first author (RBK) for his PhD thesis (p14) [37] from using information from Community Members Interested, Kathmandu [49], life expectancy, population access to electricity, national gross domestic product [47], total population Density of population [50], urbanisation (%) [51]; multidimensional poverty index (MPI) headcount [52]; per capita purchasing power [48], NMR and IMR (both reported as per 1000 live births) [53],% of disadvantaged population (further analysis by first author) based on data of Nepal Census 2012 [54], literacy rate [55], and sanctioned versus fulfilled health workers in health facilities [56]. Access to health facility refers those reported access to health facilities within 30 minutes of walking distance.

### Sampling and data

Detail sampling methodology used in NHFS is described in its original report [32]. In brief, the NHFS 2015 was a two-stage cluster survey which included the randomly selected 963 facilities proportionate to three eco-regions (Hills, Mountains, and Terai) and types of health facilites (hospitals, primary health care centers and health posts) (see detial in pages 20–21 of NHFS 2015 report). The NHFS 2015 collected information from health workers (in-charge of facilities or the most knowledgeable health workers in the facilities), pregnant women who visited facilities for the first antenatal care visit and postpartum mothers who were discharged on the day of the survey. This study included 269 facilities (which are accredited for antenatal care) for antenatal services and 109 facilities (facilities accredited as institutional childbirth services) for perinatal services where pregnant and postpartum women received those specific services, respectively. The technical quality of facilities was estimated based on the information provided by 523 pregnant women who visited facilities for their first antenatal care visit, and 309 postpartum women who were discharged on the day of the survey.

### Study variables

Independent variables included locations and types of facilities and management functions including managing authority (private, public); provinces (seven provinces); region (Mountain, Hills, and Terai); facility types (primary health care centers/ hospitals, peripheral facilities), earthquake-affected districts (affected and not affected). Other health management-related variables included quality assurance system (yes, no), frequency of facilities’ management meetings (no, sometimes, and monthly), availability of feedback collection system in facilities (yes, no), provision of external supervision of staff (yes, no), and health facility capacity (high, medium, and low).

We created a new independent variable, i.e., health facility capacity, which refers to the structural or input quality of a facility that provides a specific service. Studies reported that better input quality/ capacity of facilities could deliver better technical quality health services[29, 57–60]. Health facility capacity was created by conducting Principal Component Analysis (PCA) to generate composite-scale coefficients to reduce indicators to indices for application in comparative analyses of health facility readiness [61]. In the PCA procedure, a total of 53 items related to antenatal care services (Supplementary file, Table S2) and 73 items related to perinatal services were taken (Supplementary file, Table S3) to estimate health facility capacity for antenatal care services, perinatal services respectively. All items were then categorised into yes/no and later converted into dummy variables by assigning the value of 1 to 'yes' and 0 to 'no'. Finally, taking arbitrary cut-off point for the health facility capacity index and determined by the PCA procedure, health facilities were categorised into three equal groups: low, medium and high [62]. Two outcome variables included technical quality of health facilities for delivery of health services: i) antenatal services; and ii) perinatal services.

### Unit of analysis and outcome variables

In NHFS 2015, information was collected using the WHO's SARA framework [28]. During the pregnancy-postnatal period, women are recommended to receive several antenatal [63, 64], and perinatal care interventions [64] for the improved health status of mothers and newborns. Taking information on those interventions available in the NHFS 2015 dataset, technical quality of health facility of each outcome variable was estimated. For this, first, woman-specific technical quality of each outcome variable was estimated, then rescaled that woman-level score for each health facility level. For instance, technical quality scores of facilities were estimated using the antenatal care quality score received by each woman who attended for their first antenatal care visit. A total of 40 antenatal intervention items or procedures with pregnant women (Supplementary file, Table S4) were collected (observation of services service delivery and exit interviews) who had their first antenatal care visit [65]. The dummy value of 'yes' (= 1) if women received services, otherwise 'no' (= 0) was assigned based on the woman's response recorded in NHFS 2015. Then, the woman-specific score was calculated, taking the average of items received (out of 40). If any pregnant woman received 20 items in her first antenatal care visit, the score would be (20/40 = 0.5). Second, if any health facilities had provided antenatal services for one woman, then the woman's technical quality score would be a health facility level technical quality score for antenatal services [19]. A total of 523 pregnant women (one to four women per health facility) attended 269 health facilities for their first antenatal visit. If any health facility had more than one woman who attended for the first antenatal care visit on the day of survey, we averaged all scores of women to derive the score for that health facility.

Similarly, technical quality score of health facilities for perinatal services was calculated using the information on 24 perinatal interventions (Supplementary file, Table S5) using a similar procedure used to calculate the technical quality of health facilities for antenatal care services [65]. There were 309 postpartum women who attended 109 health facilities (one to maximum of five women per facility) for their delivery and postnatal services. If any health facility had more than one postpartum woman, we averaged the individual score of women to calculate the score for each health facility.

### Data analysis

Bivariate and multivariable linear regression analyses were conducted for each outcome variable. Descriptive statistics were reported as frequency, mean scores and proportions (%). In addition, normality was checked of the scores of the technical quality of health facilities for antenatal and perinatal services to ensure the condition to run linear regression. Technical quality scores of health facilities for both outcome variables were normally distributed. The statistical significance level was *p* < 0.05 (two-tailed). In this analysis, the linear regression coefficients (beta coefficients) were then exponentiated (log transformed) into odds ratios since the beta coefficients say very little in respect of explanation. Thus, for ease of interpretation, beta coefficients were exponentiated and reported as odds ratios [66, 67].

Before running the multivariable linear regression model for each outcome variable, multicollinearity was checked and excluded independent variables with variation inflation factor ≥ 3 [68]. The goodness of fit test was conducted using the adjusted R-squared [69] to test the model fit. First, the full regression model was run, estimated p-value for each independent variable. Then, the most insignificant variable (variable with the highest p-value) was deleted, comparing p values with other independent variables. This procedure was repeated until no insignificant independent variable was left at *p* < 0.05 [70]. Since the 2015 NHFS sample was a stratified sample, sampling weights were calculated based on sampling probabilities separately for each sampling stratum [71]. For this, the complex sampling design effect (clustering) was adjusted in the data analysis stage using the facility weight and accounting for survey strata: region and types of health facilities [71]. We have applied the sampling weights to ensure the actual representation of the survey results and provide unbiased estimates of the parameters. Further, complex sample design has been considered to adjust standard errors that accompany the properly weighted estimates. All estimates are weighted otherwise indicated. All analyses were conducted using the survey (svy) command function to adjust clustering effect in Stata 14.0 (Stata Corp, 2015).

## Results

### Descriptive characteristics of health facilities

Table 1 depicts the characteristics of health facilities providing antenatal care, and perinatal care services. Among 269 health facilities providing antenatal care services, more than two-thirds (72%) were publicly managed and did not have quality assurance mechanisms 12 months prior to the survey (76%). More than two-thirds (70%) had a monthly meeting and a feedback collection system. Of 109 health facilities providing perinatal care services, more than two-thirds (72%) were managed by the private sector. Three in four health facilities providing perinatal care services had a monthly meeting (74%) and a feedback collection system (76%).

**Table 1:** Descriptive characteristics of study health facilities providing antenatal and perinatal care services in Nepal (NHFS 2015)

Determinants	Categories	Health facilities (*N* = 269) provided antenatal services		Health facilities (*N* = 109) provided perinatal services	
		**Frequency**	**%**	**Frequency**	**%**
Managing authority	Private	38	14.3	31	28.4
	Public	231	85.7	78	71.6
Earthquake districts	Not affected	227	84.5	84	77.1
	Affected	42	15.5	25	22.9
Region	Mountain	20	7.2	13	11.9
	Hill	108	40.1	52	47.7
	Terai	142	52.7	44	40.4
Province	One	43	15.8	16	14.7
	Madhesh	51	18.8	16	14.7
	Bagmati	49	18.4	25	22.9
	Gandaki	21	7.6	12	11.0
	Lumbini	45	16.7	17	15.6
	Karnali	21	7.8	10	9.2
	Sudhur Paschim	40	14.9	13	11.9
Facility types	PHCCs and hospitals	196	72.8		
	HPs and clinics	73	27.2		
Health facility capacity	Poor	90	33.5	39	35.8
	Medium	90	33.4	34	31.2
	High	89	33.2	36	33.0
Quality assurance activities	No	204	75.7	73	67.0
	Yes	65	24.3	36	33.0
Health facility meeting	No	34	12.5	15	13.8
	Sometimes	47	17.6	13	11.9
	Monthly	188	69.9	81	74.3
Feedback collection	Yes	176	65.3	83	76.1
	No	93	34.7	26	23.9
Supervision of staff	No	57	21.2	33	30.3
	Yes	212	78.8	76	69.7

### Technical quality score of antenatal and perinatal services of health facilities stratified by independent variables

Table 2 shows the mean technical quality scores were 0.55 (maximum of 1.00) for health facilities for antenatal care as well as perinatal services. Public health facilities had low technical quality score for antenatal and perinatal services, while facilities of Madhesh province had low quality scores for antenatal and perinatal services care and Sudhur Paschim province had poor technical quality scores for perinatal care services.

**Table 2:** Technical quality scores antenatal and perinatal care services in health facilities in Nepal (NHFS 2015)

Variables	Categories	Technical quality score of health facilities for antenatal services (*N* = 269)	Technical quality score of health facilities for perinatal services (*N* = 109)
aged by	Public	0.52	0.51
	Private	0.55	0.67
Region	Terai	0.55	0.51
	Mountain	0.55	0.55
	Hill	0.56	0.60
Province	One	0.56	0.59
	Madhesh	0.52	0.45
	Bagmati	0.55	0.65
	Gandaki	0.60	0.53
	Lumbini	0.57	0.59
	Karnali	0.56	0.57
	Sudhur Paschim	0.56	0.43
Facility types	PHCCs and hospitals	0.55	
	HPs and clinics	0.56	
Health facility capacity	Poor	0.55	0.63
	Medium	0.56	0.66
	High	0.55	0.55
Health facility meeting	No	0.55	0.59
	Sometimes	0.57	0.54
	Monthly	0.55	0.55
Quality assurance activities	No	0.55	0.55
	Yes	0.55	0.56
Feedback collection	Yes	0.56	0.57
	No	0.54	0.49
Supervision of staff	No	0.54	0.53
	Yes	0.56	0.61
Mean score		0.55	0.55

### Determinants of technical quality of antenatal and perinatal services in health facilities

Table 3 shows the determinants of better technical quality of antenatal and perinatal care services in health facilities in Nepal. In the bivariable analysis, two health facility related variables (management authority and province) were significantly associated with the better technical quality of antenatal care services in health facilities. In the multivariable analysis, health facilities in Madhesh province had 4% lower odds (adjusted odds ratio (aOR) = 0.96; 95% CI: 0.92, 0.99) of providing better technical quality for antenatal care services. However, the health facilities in Gandaki province had better technical quality for antenatal care services. Conversely, of eight variables included in the regression analysis, management authority of health facilities was associated with the better technical quality of facilities for perinatal care services. Private facilities had higher odds (aOR = 1.13; 95% CI: 1.03, 1.23) of providing better technical quality perinatal care services than public facilities in Nepal.

**Table 3:** Determinants of better technical quality antenatal and perinatal care services in health facilities in Nepal (NHFS 2015)

Determinants	Categories	Quality score of facilities antenatal services		Quality score of facilities for perinatal services	
		**cOR (95% CI)**	**aOR (95% CI)**	**cOR (95% CI)**	**aOR (95% CI)**
Managed by	Public	1.00		1.00	1.00
	Private	0.96 (0.94, 0.99) ^**^		1.18(1.08, 1.28) ^***^	1.13(1.03, 1.23) ^***^
Region	Terai	1.00		0.97(0.85, 1.10)	
	Mountain	1.00(0.96, 1.04)		1.00	
	Hill	0.99(0.95, 1.03)		1.05(0.92, 1.20)	
Province	One	1.00	1.00	1.00	
	Madhesh	0.97(0.94, 1.00) ^*^	0.96(0.92, 0.99) ^*^	0.88(0.76, 1.01)	
	Bagmati	1.00(0.96, 1.03)	1.01(0.98, 1.05)	1.06(0.93, 1.21)	
	Gandaki	1.04(1.00, 1.09) ^*^	1.05(1.01, 1.10) ^*^	0.95(0.81, 1.10)	
	Lumbini	1.02(0.98,1.05)	1.01(0.98,1.05)	1.00(0.87, 1.16)	
	Karnali	1.00(0.96, 1.04)	1.01(0.97, 1.06)	0.98(0.83, 1.15)	
	Sudhur Paschim	1.00(0.97,1.04)	1.01(0.97, 1.04)	0.85(0.73, 0.99) *	
Facility types	PHCCs and hospitals	1.00			
	HPs and clinics	1.01(0.99,1.03)			
Health facility capacity	Poor	1.00		1.00	
	Medium	1.01(0.98, 1.03)		0.98 (0.89, 1.08)	
	High	0.99(0.97, 1.02)		0.90(0.82, 1.05)	
Health facility meeting	No	1.00		1.00	
	Sometimes	0.99 (0.96,1.03)		0.96(0.81, 1.12)	
	Monthly	0.98(0.95, 1.01)		0.97(0.86, 1.09)	
Quality assurance activities	No	1.00		1.00	
	Yes	1.01(0.98, 1.03)		0.99(0.91, 1.08)	
Feedback collection	Yes	1.00		1.00	
	No	0.98(0.96, 1.00)		0.92(0.83, 1.01)	
Supervision of staff	No	1.00		1.00	
	Yes	1.02(1.00,1.04)		0.93(0.85, 1.01)	
Constant			1.81(1.73, 1.89) ^***^		1.91 (1.61, 2.26) ^***^
Observations			269	109	
R-squared			0.12	0.29	

^***^ p < *0.001*

^**^ p < *0.01*

^*^ p < *0.05*

^**# **^These are separate models adjusting for covariates listed in the respective column. Variables which had *p* < 0.2 included in the final model of each outcome variable. Odds ratios were estimated after exponentiated (log transformed) β regression coefficients

^**#**^ Independent model for separate outcome variables. For antenatal care, the final multivariable linear regression explained 12.08% of the variation in health facility clinical quality for antenatal services (R-squared = 0.12). For perinatal care, the final multivariable linear regression explained 29% of the variation in health facility clinical quality for perinatal services (R-squared = 0.29)

## Discussion

In this study, we examined technical quality health facilities for antenatal care and perinatal services in Nepal. The study revealed that public and private facilities had suboptimal technical quality scores for antenatal as well as perinatal care services. However, privately managed facilities provided better quality perinatal care services than publicly managed facilities. Health facilities of Madhesh Province delivered poor quality care for the first antenatal care visit compared to other provinces.

Our study suggests the technical quality of health facilities for antenatal and perinatal services is low in Nepal. While utilisation of antenatal and perinatal services has significantly increased over the last two decades, especially regarding antenatal care and institutional delivery [72], this study indicates technical quality in public facilities for antenatal and perinatal services remain poor. This is likely to be partly because since 1991, the focus has been addressing accessibility and affordability barriers, with a limited focus on quality [73]. In recent health policies, however, health care quality has been a core component of health services with a greater focus on health infrastructure, medicines and supplies [74]. Despite increasing policy inputs for quality improvement, the current study identified suboptimal technical quality of health services. Previous studies in Nepal have also revealed poor quality of basic emergency antenatal and perinatal services, especially in physically accessible areas in eastern [75], and the southern lowland region of Nepal [76]. Poor technical quality of antenatal and perinatal services is unlikely to result in better antenatal and perinatal outcomes. This may explain why improvements in the MMR and NMR have decreased in recent years despite increased coverage.

Overall, the technical quality scores of antenatal care services were low, and health facilities of Madhesh province demonstrated significantly poorer technical quality antenatal care services than the reference province (one). In Madhesh province, several health systems issues may have influenced the access to better quality antenatal and perinatal services [58]. Although this province covers plain areas and a better transportation system than other provinces, this province has low female literacy. The majority of people speak other than the Nepali language (Maithali and Bojpuri) [37]. Studies revealed several drivers of poor health facility readiness and supply side factors contributing to poor quality services delivery in Madhesh province[76, 77]. Like Madhesh province, previous studies showed that women of Karnali province had poor access to health services and received poor quality of care from the health facilities [78–80]. Karnali province is geographically inaccessible with difficult geographic terrain and transportation system, poor health infrastructure for better health services delivery [79]. Contrarily, our analysis revealed that health facilities of Gandaki province provided better quality antenatal care services. Gandaki province has relatively high socioeconomic status, better wealth status, and good transportation facilities [48]. Previous research identified health system issues indicate inadequate health staff training, poor supervision and monitoring, and a shortage of essential medicines [76]. The health facilities' readiness score or input quality was also low in health facilities in Madhesh province [58]. Given the decentralized nature of the health system in Nepal, the municipalities of Madhesh province could implement specific strategies such as recruitment of local health workers, supplies of essential medicines, and equipment, training on technical skills, training in cultural competency.

The current study also identified better quality perinatal services in private health facilities than public health facilities. This difference may be because most private facilities are in urban areas; they are likely to have better infrastructure and equipment. Private facilities employ trained and better qualified healthcare workers as much of their workload relates to inpatient care [81]. Evidence suggests private facilities in Nepal have better management practices than public facilities, especially the people management component is significantly better among private hospitals [82]. Better management functions are associated with the indicators of the performance of the hospitals that may be the reason for the better quality of care in private facilities [83, 84]. Private facilities also tend to have higher rates of caesarian section delivery [85, 86], than public services, which is likely to increase the inpatient services, and postnatal care post cesarean section delivery. For example, in the case of caesarian delivery, postpartum mothers and newborns are more likely to be examined following all technical procedures before getting discharged from facilities [87]. Private facilities usually keep women in hospitals longer post-delivery than public facilities, which may be a reason for better quality perinatal care services. However, women attending private facilities for maternity services, usually incur out-of-pocket expenditure for all inpatient care after getting perinatal services. The high cost of care in private facilities means women from the most socioeconomically disadvantaged populations cannot afford routine antenatal and perinatal services in private facilities due to the out-of-pocket expenditure. Even where disadvantaged women are enrolled in the national health insurance scheme this does not extend to private facilities. Women from disadvantaged backgrounds therefore are more likely to receive antenatal and perinatal services in public facilities where the quality of care is suboptimal [53]. This is also reflected in the MMR and NMR, with women from disadvantaged populations having a higher MMR and NMR than those from more privileged backgrounds [53]. The government’s Safe Delivery Incentive Program (SDIP) aims to address the demand side financing barriers, especially for women from socioeconomically disadvantaged groups, by subsidising the cost of maternity care. All women receive a financial incentive if they complete recommended four antenatal care visits and give birth at facilities through this program. The SDIP, however, is mainly implemented through public facilities and only a few private facilities have implemented the SDIP [39]. Scaling-up the SDIP to all facilities irrespective of their management (public or private), would allow women to access private health services at subsidised costs.

Besides improving access to private health services, there is an urgent need to improve in quality of care in public facilities. In Nepal, women with low socio-economic status and remote areas visit public facilities more vulnerable to maternal and newborn mortalities than other women who can afford private health facilities. For instance, about two-thirds of childbirth occur in public facilities, mostly in birthing centers located in peripheral areas [39]. Therefore, health systems efforts need to focus on better health facility inputs in relation to infrastructure, equipment, and trained human resources. Additionally, some management-related variables such as supervision and ensuring quality assurance activities health facilities, onsite coaching of health workers can improve the quality of public health facilities. Previous evidence from Nepal suggests onsite supervision of peripheral health workers improves technical capacity and management of health facilities [88]. In addition, studies in other LMICs have also documented suboptimal quality of care delivery due to a lack of clinical audits and feedback, or performance-based incentives, and inadequate supportive supervision of staff at the local level health facilities [89, 90]. Thus, health system efforts need to focus on supervision, feedback mechanisms and quality assurance activities and onsite coaching and mentoring health workers in peripheral health facilities.

### Implications for programs and policies

This study has some implications for programs and policies. First, this study suggests the need to focus on the delivery of technical quality antenatal and perinatal services in Madhesh province. Local governments of Madhesh province should design and implement contextual and targeted programs. The federal government and provincial governments should work with multiple local level stakeholders, especially in implementing public health policies and making services available for disadvantaged populations. The delivery of quality antenatal and perinatal services through peripheral health facilities is important to reduce perinatal deaths in Nepal. Provincial and local governments need to focus resources on improving the health facility readiness for better quality health services in public facilities. Most marginalised communities of rural areas seek care from public facilities, who are most vulnerable to maternal and neonatal morbidities and moralities. For access to private maternity services, policymakers could formulate multi-pronged strategies; first, there is needed to strengthen public facilities. Second, strategies for private sector engagement may include regulating the private sector and monitoring of private facilities, and designing a regulatory and health governance framework for private facilities. At the same time, private facilities should be regulated whether poor women are getting services in private facilities as part of corporate social responsibility [91]. Third, public policies need to be implemented in private facilities including SDIP or national health insurance program could increase the access of disadvantaged women in Nepal. The methods employed in this research could also be of relevance to decision-makers interested in assessing the technical quality of facilities for antenatal and perinatal services in other low-and lower-middle-income countries.

### Strengths and limitations

As with all studies, this study has some strengths and limitations. Strength is information obtained from interviews with health workers, health facility inventory, clients who got services, and information obtained from providers and client’s interactions. This study is nationally representative so that findings can be generalised at the national and province levels. Limitations include the NHFS 2015 was conducted more than five years ago, and the findings could represent the status around that time. However, due to the lack of recent data, the study used the most available data to assess the delivery of quality antenatal and perinatal services from facilities. So far, another round of health facility survey data has not been released; the evidence presented in this analysis can be interpreted as a baseline for future studies. The NHFS was cross-sectional and observational data, the findings of this study. Thus, the association presented in the findings represent correlations rather than causality. Finally, the study did not provide the users' experience in the care provided by health facilities, which is an important dimension in the quality of care.

Nevertheless, the study provides important insights into the objective assessment of process quality of care of antenatal and perinatal services. It demonstrates how multiple data sources can be used to create a technical quality score for health facilities of antenatal and perinatal services. In this study, some management related variables such as supervision of staff, feedback mechanism or quality assurance activities were insignificant in the multivariable analysis. In addition, some independent variables (e.g., urban/rural, client flow in health facilities) might be relevant to include in the analysis, but we were unable to include in the analysis due to data limitations. Furthermore, this study analysed secondary data, which restricted the use of information available in the dataset. Further studies with a large sample size can be conducted to address these data limitations.

## Conclusions

Health facilities had suboptimal technical quality scores for antenatal and perinatal services. Health facilities of Madhesh province had poor technical quality for antenatal services; privately managed facilities provided better technical quality for perinatal services than public facilities. Provincial and local governments of Madhesh province need to work to upgrade the quality of care in public facilities. Local governments need to increase the resources on health system readiness (e.g., ensuring trained local human resources, equipment, medicine, building), regular supervision and monitoring, and timely feedback on local problems to improve the quality of public health facilities. In the context of Nepal's federalised health system governance, municipal governments may utilise resources to enhance the quality of care in health facilities, including health workforce recruitment, infrastructure development, and arranging equipment and supplies of essential medicine. The federal government should develop a regulatory framework to improve access of routine health services in private facilities without financial hardship targeting disadvantaged groups. The implementation of SDIP in private facilities could also increase the access to private health services, including antenatal and perinatal services.

## Supplementary Information

**Additional file 1:** **Table S1.** Health policy trajectory in the last three decades (1990-2019) in Nepal. **Table S2.** Items included in the HF capacity assessment for antenatal care services. **Table S3.** Items included in the HF capacity assessment for perinatal services. T**able S4.** Items for the assessment of the technical quality of HFs for antenatal care services. **Table S5.** Items for the assessment of the technical quality of HFs for perinatal services.

## Data Availability

Data used in this study are publicly available secondary data obtained from the DHS (https://dhsprogram.com/data/available-datasets.cfm) program.

## Abbreviations

LMICs: Low and lower-middle-income countries
MNH: Maternal and newborn health
SDIP: Safe Delivery Incentive Program
NHFS: Nepal Health Facility Survey
SARA: Service Availability and Readiness Assessment
SDG: Sustainable Development Goals
PHCC: Primary health care centres
HP: Health post
