# Supplementary material for: Investigation of technical quality of antenatal and perinatal services in a nationally representative sample of health facilities in Nepal
Source: Arch Public Health. 2022 Jul 4;80:162. doi: 10.1186/s13690-022-00917-z (PMC9252055; doi:10.1186/s13690-022-00917-z)
Supplement: Supplementary file 1 — Additional file 1: Table S1. Health policy trajectory in the last three decades (1990-2019) in Nepal. Table S2. Items included in the HF capacity assessment for antenatal care services. Table S3. Items included in the HF capacity assessment for perinatal services. Table S4. Items for the assessment of the technical quality of HFs for antenatal care services. Table S5. Items for the assessment of the technical quality of HFs for perinatal services. [file 13690_2022_917_MOESM1_ESM.doc]

## Supplementary file 1

Table S1: Health policy with focus on MNH policy reform trajectory in the last three decades (1990-2019) in Nepal

| Document category | 1990-1999 | | 2000-2009 | | 2010-2019 | |
| --- | --- | --- | --- | --- | --- | --- |
| Constitutions | 1991 | Constitutional Monarchy and Multiparty Democracy Constitution of Nepal | 2006 | The interim constitution of Nepal | 2015 | Federal republic constitution of Nepal |
| Laws acts and regulations | 1992 | Mothers Milk Substitutes (Control of sale and distribution) Act, 1992 | 2002 | 11th amendment of Civil code (permission of safe abortion) | 2018 | Safe Motherhood and Reproductive Health Rights Act |
|  | 1995 | Nursing Council Act  Health Professional Council Act |  |  | 2015 | Immunisation Act |
|  | 1996 | Iodised salt (production and distribution) act. Nepal Health Service Act | 2009 | Health workers and health facilities protection act Governance (Management and Implementation) Act,  Governance (Management and Implementation) Regulation | 2016 | Reproductive health rights act |
|  | 1999 | Local Self Government Act,  Local Self Government Regulation  Iodised salt (production and distribution) act |  |  | 2017 | Public Health Act. Local Government Operation Act,  Health Insurance Act**.**  Health Insurance Regulation  Public Health Service Regulation  Civil Code 2017 (Decriminalise Chhaupadi practices)  Procurement improvement plan 2017-2021 |
|  |  |  |  |  | 2018 | Safe Motherhood and Reproductive Health Right Act  Immunisation Regulation 2074 |
|  |  |  |  |  | 2020 | Public Health Service Regulation |
| Policies strategies, plans | 1997 | Second long-term health plan  Nepal reproductive health policy | 2000 | National adolescent and development health strategy | 2010 | Nepal Health Sector Implementation Plan -2 |
|  | 1991 | National health policy | 2002 | National Strategy for control of anaemia among women and children | 2012 | Female  Community Health Volunteers revised strategy |
|  | 1994 | Subhealth post established  Primary health care outreach clinics introduced  Safe Motherhood Plan of action | 2003 | National Safe Abortion Policy  Local Self Governance (First Amendment) Ordinance.  Poverty Alleviation Ordinance.  National Safe Abortion Policy.  National Health Research Policy of Nepal.  Strategic Plan for Human Resources for Health.  National Guidelines for Counselling, Testing and Referral | 2013 | Multi-sectoral Nutritional Plan (MSNP) |
|  | 1996 | National maternity care guideline | 2003 | National Neonatal Health Strategy  Health Sector Strategy: An Agenda for Reform.  Nepal Health Sector Programme - Implementation Plan  Sector Wide Approach (SWAp) adopted.  National Neonatal Health Strategy.  Nepal Red Cross Society Health Policy  National nutrition policy and strategy | 2014 | Every newborn Action plan implementation plan of action  National health insurance policy 2014  National Health Policy 2014 |
|  | 1998 | Nepal maternal morbidity and mortality survey | 2006 | National Policy on Skilled Birth Attendant  Safe Motherhood and Neonatal health long term plan (SMNHLTP 2006-2017)  School Health and Nutrition Strategy  National Blood Transfusion Policy (revised)  Free health care program | 2015 | Nepal social health insurance program  Nepal Health sector strategy (2015-2020) |
|  |  |  | 2007 | Free health care policy  Policy on Quality Assurance in Health Care Services |  |  |
|  |  |  | 2009 | National Nutrition Policy and Strategy Updated  Gender equity and social inclusion strategy (GESI)  Remote areas guideline | 2016 | National Strategy for Reaching the Unreached (2016-2030)  Basic Health Care strategy |
|  |  |  |  |  | 2017 | National E-health strategy  Health Insurance Act  Partnership Policy in Health |
|  |  |  |  |  | 2018 | National Adolescent Health and Development Strategy  Gender equity and social inclusion in the health sector (revised) |
|  |  |  |  |  | 2019 | 15th 5-year plan. Health Insurance Regulation  National Health Policy |
| Programs projects/surveys | 1995 | Community-based ARI and control of diarrheal diseases | 2000 | Child survival and health grant program  State of world newborn launched | 2010 | Comprehensive multi-year plan of action in immunisation  Community Based Newborn Care Package (CBNCP)  Maternal and perinatal deaths surveillance and response  Rereferral funds to hospitals where comprehensive obstetric and neonatal care services are not available |
|  | 1996 | Nepal family health survey | 2001 | Nepal family health project  Saving newborn lives program, I  NDHS  State of world newborn health- Nepal report | 2011 | Nepal Health Sector Support Program (NHSSP)  Integrated Nutrition program (SUAAHARA)  Nepal demographic and health survey  Navi Care Program  The rural ultrasound scan program  Chlorhexidine introduced |
|  | 1997 | Nepal safe motherhood project  community-Based Integrated Management of Childhood Illness (CB-IMCI) | 2002 | National Safe Motherhood Plan (2002-2017)  Birth Preparedness Package Program introduced through Female Community Health Volunteers  Abortion legalised | 2012 | Infant and Young Child Feeding (IYCF) and Growth Monitoring piloted. Saving newborn lives program III  MNH readiness hospital and birthing center/basic emergency obstetric and neonatal care quality improvement |
|  | 1998 | Decentralised action for children and women (DACAW)  Iron and Folic Acid (IFA)  National rollout of Female Community Health Volunteers program  Supplementation for pregnant and lactating women.  Nepal micronutrient survey | 2003 | Intensification of Maternal and Neonatal Micronutrient Program (IMNMP)  Birth Preparedness Package and MNH activities at the community level | 2013 | Nyano Jhola (Warm Bags) Program  Maternal and perinatal death surveillance and response guideline  onsite clinical coaching /mentoring programme  PNC home visit (microplanning for PNC)  Calcium during ANC piloted |
|  |  |  | 2004 | Deworming with single dose of Albendazole  Safe abortion program (surgical) | 2014 | Community-Based Integrated Management of Neonatal and Childhood Illness (CBIMNCI); Verbal Autopsy survey; Multiple Indicator Cluster Survey (MICS)  Facility based IMNCI program |
|  |  |  | 2005 | Community Based Maternal Newborn Health Program (CBMNH)  Inclusion of Zinc in ORS piloted | 2015 | Free newborn care program  Integrated nutrition program (SUAAHARA II)  Nepal health insurance program  Nepal Health Facility Survey (NHFS)  Emergency referral funds |
|  |  |  | 2006 | CBIMCI plus newborn care  Child Protection Grant with Infant and Young Child Feeding Counselling and food supplementation  Saving Newborn Lives program II  Nepal demographic and health survey  Nepal Family Health Project II  Maternity incentive program, (financial incentives to pregnant women cover transport cost) | 2016 | Nepal demographic and health survey  Reaching the unreached strategy (2016-2030) Aama and free newborn care programme |
|  |  |  | 2007 | Safe delivery incentive program added incentives for health workers and free institutional delivery care Chlorhexidine (CHX) Cord Care program  Technical Working Group (National Plan of Action) for newborn.  Free health care program  skilled birth attendants Policy- task shifting with MBBS doctors performing C-sections, and nurses, and anesthesia assistants | 2018 | Strengthening systems for better health (SSBH)program  GESI Operational guideline |
|  |  |  | 2009 | Remote areas guideline for safer Motherhood  Aama program – four ANC and transport incentives, free delivery, and institutional reimbursement. Misoprostol distribution through Female Community Health Volunteers.  Local recruitment of comprehensive obstetric and neonatal care teams  Nutrition Assessment and Gap Analysis (NAGA)  Maternity Incentive Program  Community-Based Newborn Care package (CB-NCP)  • Scaling Up Nutrition (SUN  • Pilot study of VAS of newborn (4 districts)  • Piloting of Micro-nutrient powder (MNP  • Piloting of Community-based Management of acute malnutrition (CMAM)  CBNCCP introduced | 2019 | Multiple Indicator Cluster Survey (MICS) |
| Sources: Prepared by the first author for [1] (p:171-173), using multiple sources of information Policy review of Aama program [2], Success factors of MDGs[3], Annual health report 2019 [4]. Stocktaking the health policies of Nepal [5], a systematic review of universal health coverage in Nepal [6], Nepal Safe Motherhood and Newborn Health Roadmap 2030 [7]. | | | | | | |

##

Table S2: Items included in the HF capacity assessment for ANC services.

| **Status of general readiness** | **Yes % (N=269)** | **Availability of equipment** | **Yes % (N=269)** |
| --- | --- | --- | --- |
| 24-hour staff availability | 54.9 | Water for infection prevention | 68.7 |
| Water supply | 92.4 | Soap for Infection Prevention (IP) | 72.5 |
| Client latrine | 94.1 | Disinfectant for IP | 66.8 |
| Emergency transport | 82.3 | Autoclave services | 98.7 |
| Client waiting area | 94.2 | Digital blood pressure (BP)apparatus | 3.0 |
| Landline phone | 55.2 | BP set manual | 95.7 |
| Electricity service | 88.1 | Stethoscope | 97.6 |
| **Availability of ANC services** |  | Examination light | 56.1 |
| Iron tablet distribution | 74.5 | Fetoscope available | 95.7 |
| Folic acid distribution | 63.4 | Weighing scale | 98.9 |
| Tetanus toxoid service | 94.1 | Exam table | 98.3 |
| Albendazole tablets distribution | 96.3 | Tape fundal height | 44.0 |
| Misoprostol distribution | 23.1 | Thermometer | 78.6 |
| Urine test service | 35.4 | **Availability of medicine** |  |
| Haemoglobin test services | 31.9 | Misoprostol tablets | 36.4 |
| ANC counselling | 99.6 | Iron-folic tabs | 71.8 |
| Birth preparedness package counselling | 99.0 | Tetanus toxoid vaccine | 43.0 |
| Family Planning counselling | 95.8 | Albendazole tablets | 84.8 |
| Counselling on prevention of HIV/AIDS | 94.9 | **Staff training and guidelines** |  |
| Breastfeeding counselling | 95.9 | ANC guideline | 27.0 |
| Newborn care counselling | 95.2 | IEC materials for ANC | 75.3 |
| PNC counselling | 96.0 | Supervision | 18.6 |
| Weighting clients | 92.0 | ANC screening | 18.6 |
| Measure BP services | 93.1 | ANC counselling | 20.9 |
| Health education service | 21.0 | Complication and management | 20.9 |
| Urine portion test | 44.8 | Nutritional assessment | 13.0 |
| Anaemia test services | 53.4 | Other training | 4.1 |
| HIV test and counselling | 55.9 |  |  |
| Measurement of height | 34.7 |  |  |

Table S3: Items included in the HF capacity assessment for delivery and postnatal services.

| **Availability of equipment** | **Yes, % (N=109)** | **Status of general readiness** | **Yes, % (N=109)** | **Availability of medicines** | **Yes, % (N=109)** |
| --- | --- | --- | --- | --- | --- |
| Heat source available | 82.2 | Landline phone | 93.0 | Injectable antibiotic | 84.2 |
| Examination light | 96.1 | Mobile phone | 26.7 | Tablet oxytocin | 99.2 |
| Dee-lee suction | 56.8 | Water supply | 96.1 | Tablet magnesium sulphate | 91.4 |
| Bag and Mask | 98.4 | Client latrine | 96.1 | Intravenous fluid | 98.4 |
| Thermometer | 92.1 | Protected client waiting area | 96.1 | Betadine solution | 96.8 |
| Infant scale | 99.2 | Electricity service | 96.9 | Chlorhexidine tube | 47.3 |
| Fetescope | 95.9 | Emergency transport | 94.6 | Calcium gluconate | 76.0 |
| Blood pressure set | 96.8 | 24 Hr duty call for delivery services | 80.2 | Nifedipine capsule | 62.0 |
| Stethoscope | 98.4 | **Staff, guideline, and training** |  | **Availability of newborn services** |  |
| Delivery bed | 99.2 | IMPAC training | 40.3 | Newborn resuscitation | 87.0 |
| Delivery set | 97.6 | Routine labor and delivery | 34.8 | Kangaroo mother care | 85.7 |
| Cord clamper | 92.9 | AMTSOL | 32.7 | Injectable antibiotic available | 84.2 |
| Vaginal speculum | 94.4 | MNH update emergency obstetric care | 28.0 | Skin to skin contact | 88.5 |
| Cord cutting blade | 96.1 | Neonatal resuscitation training | 33.7 | Wrapping baby | 100 |
| Epitomy set | 96.1 | Exclusive breastfeeding training | 28.9 | Immediate breastfeeding | 98.4 |
| Suturing blade | 98.3 | Neonatal sepsis management | 21.8 | Head to toe | 98.4 |
| Needle holder | 96.8 | Thermal care | 24.2 | Weighing newborn | 100 |
| Forceps | 91.3 | Cord cutting training | 28 | Use of chlorhexidine | 53.6 |
| Sponge holder | 96.8 | Kangaroo Mother Care training | 31.2 | Delayed bathing | 70.5 |
| Blank paratograph | 82.2 | Other training | 2.4 | **Availability of delivery services** |  |
| Baby wrappers four sets | 86.5 | Supervision Health worker | 97.7 | Antibiotics parental | 91.4 |
| Nayano Jhola set | 40.9 | External supervision in the last four months | 70.5 | Oxytocin parental | 98.4 |
| Water available in the delivery room | 95.3 | Reproductive health guideline | 27.7 | Anticonvulsant parental | 52.1 |
| Soap available in the maternity room | 94.5 |  |  | Injectable antibiotic | 84.2 |
| Alcohol for hand rub in the maternity room | 45.6 |  |  | Use of paratograph | 85.4 |
| Latex gloves | 97.6 |  |  |  |  |
| Disinfectant available in the maternity room | 92.9 |  |  |  |  |
| Autoclave services | 100 |  |  |  |  |

Note IMAC: Skilled birth attendant, Integrated management of pregnacy and childbirth. AMTSOL: Acute management of the third stage of labour.

Table S4: Items for the assessment of the technical quality of HFs for ANC services.

| **ANC interventions** | **Yes (N=523), %** |
| --- | --- |
| Asked clients age | 53.6 |
| Mensuration date asked | 25.4 |
| The previous history asked | 100 |
| Asked veginal bleeding | 100 |
| Asked for eye vision | 100 |
| Checked swelling | 100 |
| Foetal measurement | 100 |
| Physical examination | 100 |
| Measured Blood Pressure | 100 |
| Weight took | 85.3 |
| Palm checked | 62.1 |
| Checked for oedema | 42.9 |
| Palpated abdomen | 36.0 |
| Checked breast | 22.5 |
| Listen to foetal heartbeat | 83.6 |
| Tested haemoglobin | 50.2 |
| Tested blood grouping | 47.9 |
| Counselling on urine test | 100 |
| Counselling on syphilis test | 25.2 |
| Counselling on HIV test | 100 |
| Counselling health pregnancy | 24.3 |
| Nutritional counselling | 52.9 |
| Counseling on iron prophylaxis | 100 |
| Counselling on deworming | 49.8 |
| Tetanus Toxoid counselling | 42.6 |
| Institutional delivery promotion | 100 |
| Newborn postpartum recommendation | 100 |
| Advise dangers signs during pregnancy | 100 |
| Advise dangers signs during delivery | 100 |
| Advise dangers signs of newborns | 100 |
| Advise danger signs during postpartum | 100 |
| Received iron | 56.2 |
| Nutritional counselling | 52.4 |
| Pregnancy complication counselling | 22.7 |
| Advise what to do after complication | 100 |
| Advise preparation for delivery | 9.6 |
| Plan for delivery | 17.2 |
| Counselling for exclusive breast feeding (EBF) | 8.1 |
| Family planning counselling | 4.2 |
| Advise EBF within one hour | 6.3 |

Table S5: Items for the assessment of the technical quality of HFs for delivery and postnatal services.

| **Delivery and postnatal interventions** | **Yes (N=309), %** |
| --- | --- |
| Measured Blood Pressure | 81.0 |
| Checked pulse | 72.9 |
| Checked temperature | 57.3 |
| Checked swelling | 43.3 |
| Checked perineum | 61.5 |
| Checked breast | 43.3 |
| Asked for urine | 63.4 |
| Checked uterine | 56.7 |
| Asked if any bleeding | 67.3 |
| Cord care | 54.4 |
| Breast Feeding advise | 82.6 |
| Family Planning advise | 25.1 |
| PNC advise | 63.3 |
| Would situation examination | 49.0 |
| Advise on dangers signs | 38.2 |
| Checked baby's temperature | 67.6 |
| Checked baby's breathing status | 64.4 |
| Checked colour and movement | 58.4 |
| Cord examination | 58.9 |
| Checked skin | 32.0 |
| Checked eyes | 36.2 |
| Checked for Jaundice | 47.8 |
| Asked on breastfeeding situation | 84.7 |
| Asked on immunisation situation | 64.8 |

**References**

1. Khatri RB: **Towards equity of maternal and newborn health services in Nepal**. *PhD Thesis.* The University of Queensland, Australia; 2021.

2. Khanal GN: **Conditional cash transfer policies in maternal health service utilization in Nepal: Analysis of safe delivery incentive program (Aama Surakshya Karyakram) using Kingdon's multiple streams framework**. *The International Journal of Health Planning and Management* 2019, **34**(1):e131-e141.

3. MOHP [Nepal]: **Success Factors for Women’s and Children’s Health: Multisector Pathways to Progress**. In*.*: Ministry of Health and Population, Kathmandu Nepal.; 2015.

4. MOHP [Nepal]: **Annual Report, Department of Health Services 2075/2076 (2018/2019).** In*.*: Ministry of Health and Population. Kathmandu, Nepal. Ministry of Health and Population, Department of Health Services.; 2020.

5. NHSSP M: **Report on Stocktaking the Health Policies of Nepal**. In*.*: Nepal Health Sector Support Programme III; 2018.

6. Ranabhat CL, Kim CB, Singh A, Acharya D, Pathak K, Sharma B, Mishra SR: **Challenges and opportunities towards the road of universal health coverage (UHC) in Nepal: a systematic review**. *Arch Public Health* 2019, **77**(1):5.

7. FWD: **Nepal Safe Motherhood and Newborn Health Road Map 2030**. In*.* Kathmandu Ministry of Health and Population, Government of Nepal,; 2019
